# The Concept of Postbiotics

**DOI:** 10.3390/foods11081077

**Published:** 2022-04-08

**Authors:** Gabriel Vinderola, Mary Ellen Sanders, Seppo Salminen

**Affiliations:** 1 Instituto de Lactología Industrial (INLAIN, UNL-CONICET), Facultad de Ingeniería Química, Universidad Nacional del Litoral, Santa Fe 3000, Argentina; 2 International Scientific Association for Probiotics and Prebiotics, Centennial, CO 80122, USA; maryellen@isappscience.org; 3 Functional Foods Forum, Faculty of Medicine, University of Turku, 20014 Turku, Finland; sepsal@utu.fi

**Keywords:** postbiotics, inanimate, probiotics, International Scientific Association for Probiotics and Prebiotics, ISAPP, non-viable probiotics, heat-inactivated probiotics, microorganisms

## Abstract

The scientific community has proposed terms such as non-viable probiotics, paraprobiotics, ghostbiotics, heat-inactivated probiotics or, most commonly, postbiotics, to refer to inanimate microorganisms and/or their components that confer health benefits. This article addresses the various characteristics of different definitions of ‘postbiotics’ that have emerged over past years. In 2021, the International Scientific Association for Probiotics and Prebiotics (ISAPP) defined a postbiotic as “a preparation of inanimate microorganisms and/or their components that confers a health benefit on the host”. This definition of postbiotic requires that the whole or components of inactivated microbes be present, with or without metabolic end products. The definition proposed by ISAPP is comprehensive enough to allow the development of postbiotics from different microorganisms, to be applied in different body sites, encouraging innovation in a promising area for any regulatory category and for companion or production animals, and plant or human health. From a technological perspective, probiotic products may contain inanimate microorganisms, which have the potential to impart a health benefit. However, their contribution to health in most cases has not been established, even if at least one probiotic has been shown to confer the same health benefit by live or inanimate cells.

## 1. Introduction

Cell viability has long been regarded as important for a probiotic to confer a health benefit. However, it has been long recognized that non-viable microbes, their cell components, and their metabolites also can impact health. A number of different terms can be found in the published research to address these preparations: non-viable probiotics, heat-killed probiotics, tyndallized probiotics, cell lysates, paraprobiotics, ghostbiotics and postbiotics [1]. In mid-2021, a definition of postbiotics was proposed by the International Scientific Association of Probiotics and Prebiotics (ISAPP), stating that a postbiotic is a “preparation of inanimate microorganisms and/or their components that confers a health benefit on the host” [1]. This definition was debated for more than one year by a consensus panel composed of scientists with different backgrounds and perspectives. Usage of this term has grown over recent years, but until 2021, no consensus definition had been published, and indeed, many different conceptualizations of this term were in use. This precise, well-considered definition provides a basis for clarity and accuracy in communications about postbiotics, to the benefit of scientists, industry and regulators. This paper discusses the consensus term ‘postbiotic’, including the rationale behind the definition, and explores their technological development and future use in foods.

## 2. Postbiotic Definition

Postbiotic is a term derived from the Greek for ‘post’, meaning after, and ‘bios’, meaning life. Further, the ‘biotic’ family of terms (probiotics, prebiotics, synbiotics and postbiotics) coalesces around microbes (or their substrates) (Table 1). Therefore, the term postbiotic appropriately refers to substances derived after the microorganisms are no longer alive, or, in other words, inanimate, dead or inactivated. The microbes comprising a postbiotic may be inanimate, intact cells or may be structural fragments of microbes, such as cell walls. Many preparations of postbiotics also retain microbe-produced substances, such as metabolites, proteins, or peptides, which may contribute to the overall health effect conferred by a postbiotic, but such components are not essential to a postbiotic. A postbiotic must be derived from a well-defined microorganism or combination of microorganisms for which genomic sequences are known and prepared using a delineated technological process of biomass production and inactivation, which can be reliably reproduced.

Prior to settling on the definition of postbiotic, the ISAPP panel considered other definitions that had been previously published (Table 2), as well as the Greek derivation of the word. Most published definitions of postbiotic focused on metabolites or factors produced by microbes. Some also included dead or inactivated microbes. Other stipulations in some definitions were problematic. For example, the requirement that a postbiotic should be produced from a probiotic places capricious restrictions on a postbiotic. A probiotic by definition must meet specific criteria, including a documented health benefit [9]. It is not clear what the value is of stipulating that the starting material for a postbiotic must in itself be proven to be a probiotic, when a postbiotic is so different from a probiotic and an established health benefit for a live microbe does not predict a benefit in an inactivated form. Such a definition would also be a barrier to innovation as the research path should first establish that a microbe is a probiotic, or restrict the starting microbe to already established probiotics, and then go on to meet the criteria for a postbiotic when the latter should be sufficient. Further, definitions that focused on metabolites from probiotics were problematic as, by that definition, a metabolite produced by a probiotic could be a postbiotic, whereas the chemically identical metabolite produced by a microbe not established to be a probiotic would not be. Another concern about some previous definitions is the absence of a clear requirement for a health benefit. If no health benefit is specified, then it is uncertain what value is added by the use of a postbiotic. Some definitions were unclear if the postbiotic was to be administered to a target host or could instead be produced in situ via the normal activities of resident or administered microorganisms. Definitions that do not distinguish between a product that is administered and substances that are produced in situ have an unclear path to translation into foods, feeds and other final products. Finally, other definitions stipulated that postbiotics target only the gut lumen [10], leaving out the possibility of applying postbiotics to other surfaces, such as the vagina or the skin. Taken together, the previously published definitions of postbiotic were deemed to be vague and lacking consideration of important, practical factors. Further, as discussed later, exciting new research in the area of potential health benefits of inactivated microbes has accumulated. Hence, the ISAPP consensus panel focused on the inanimate microbe, not on their metabolic outputs. Opposition to the ISAPP definition was levied shortly after its publication [11], and the corresponding reply refuting the criticism was also published [12].

## 3. How Postbiotics May Impact Our Vision about Probiotics for Future Research

**Figure 1:**
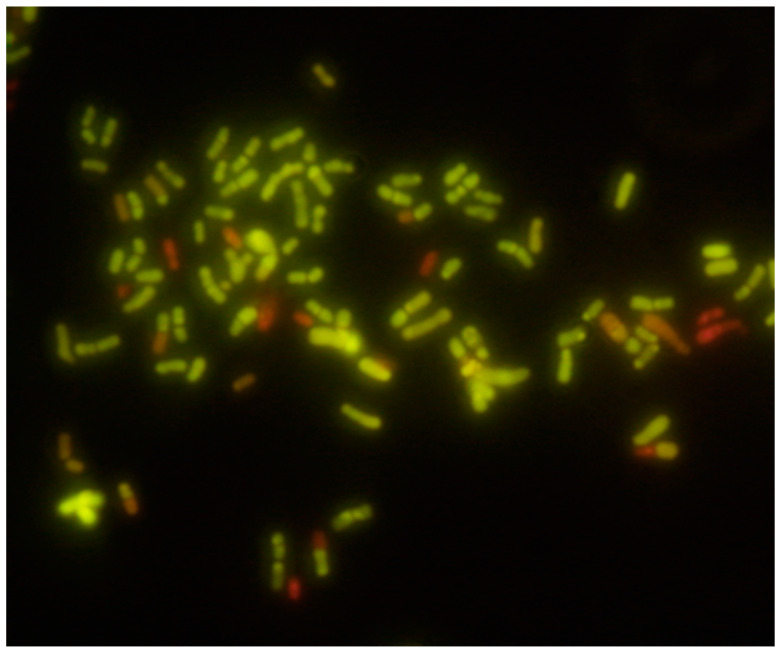
*Bifidobacterium animalis* subsp. *lactis* INL1 seen under fluorescence microscopy (1000× magnification) after staining with a cell viability probe. Live cells are stained in green, whereas non-viable cells are shown in red. An overnight (18 h, 37 °C, anaerobiosis) culture of the strain was stained with LIVE/DEAD^®^ BacLight™.

Probiotic foods and food supplements should be formulated to confer the expected health benefit. This requires the product to retain efficacious levels of live microbes in the product through the end of its shelf life. However, live cells coexist with non-viable cells, even in a fresh, overnight culture of the strain (Figure 1), but potentially to an even greater extent at the end of shelf life.

In fact, cells that go unseen by traditional agar plating used to enumerate viable cells in probiotic products, such as viable but not culturable cells (VBNC) or dead cells, may constitute a major proportion of a probiotic culture, and this proportion likely varies according to many factors, including the pH and phase of growth of the culture prior to harvesting. Cultures harvested in the late stationary phase typically contain a larger proportion of non-viable cells than in the early stationary phase or in the exponential phase, and cultures grown at pH close to neutrality contain a larger proportion of non-viable cells than those grown at a lower pH [18]. Non-viable cells can be observed through fluorescence microscopy or flow cytometry [19]. Using flow cytometry, researchers demonstrated that a fresh culture of *Lacticaseibacillus rhamnosus* R001 contained a 100:1 ratio of live:dead cells, where just after freeze drying, the number of dead cells significantly increased to render almost a 1:1 ratio [20]. Taking into account that some loss of cell viability occurs during the manufacture of probiotic cultures and during the shelf life of the product, manufacturers usually overfill the products with live cells beyond the declared dose to assure adequate levels of live cells at the end of the product shelf-life. Overfilling can range from 1.5 to 4 times the number of live cells expected by the end of the shelf life, depending on the strain, dosage form and packaging [21]. However, there is a scarcity of published data about industrial overfilling practices [21]. In a completely theoretical scenario, a probiotic food supplement that is expected to deliver, for example, 60 billion CFU, and which was verified during development to lose 75% viability by the end of shelf life, should be overfilled to 240 billion CFU at manufacture. By the end of its shelf life, it would still be able to deliver 60 billion live cells, but also 180 billion inanimate cells. The implications of this high level of non-viable cells in commercial products have not been addressed in the published literature. Can we consider the 180 billion non-viable cells delivered in such a product to be postbiotics? The answer is ‘no’, primarily because in order to be a postbiotic, those non-viable cells themselves must be shown to confer a health benefit. Further, the product as described remains a probiotic as it is able to deliver an efficacious dose of live microorganisms.

However, one may still hypothesize that inanimate cells can participate in the overall health benefit expected for a probiotic product. This hypothesis can be made on the basis that some strains, and for some endpoints, deliver similar benefits as viable and non-viable microbes [8,22,23]. Since the probiotic definition does not exclude inanimate microbes, and since it is technologically infeasible to rid a probiotic of inanimate cells, the significance of their presence in probiotic products deserves investigation. Such research is complicated by the fact that a probiotic product may have variable and significant amounts of inanimate cells. The challenge is to understand what would be the potential contribution of inanimate cells to the health benefit as, for instance, they can still display the surface antigens of a live cell, which could be involved in the mechanism of action that supports the health benefit.

The accumulation of inanimate cells over the shelf life of a probiotic food supplement may be interesting to consider in future efficacy trials where long-term administration of the probiotic takes place. For example, in studies of 12 months’ duration, it is rarely reported if the same batch of the probiotic supplement was used, or if different fresh batches were supplied and with what frequency [24]. The level of non-viable cells these products contain over the duration of the trial are mostly not reported. The design of future clinical trials to collect and report these data will help us to better understand the contribution of inanimate microbes to the health effects conferred by probiotics.

## 4. Characteristics of Postbiotics as They Relate to Foods

Probiotics are live microorganisms that, when administered in adequate amounts, confer a health benefit on the host [3]. Probiotics have been included in a variety of products: fermented (yoghurt, cheese) or non-fermented (cereal or chocolate bars, fruit juices, smoothies) foods, which can be of dairy origin or plant-based, with yoghurt (or fermented milks in general) perhaps the food most commonly used for the delivery of probiotics [25]. However, certain food characteristics (acidity, water activity, specific chemical compounds) or storage conditions (moisture, temperature, package permeability to oxygen, time) may impose challenges to the proper survival of probiotics in foods during production and storage [16]. In this context, the unsatisfactory survival of certain probiotic strains in some foods has been systematically reported [26]. Therefore, inanimate microbes are also likely components of many food products.

**Figure 2:**
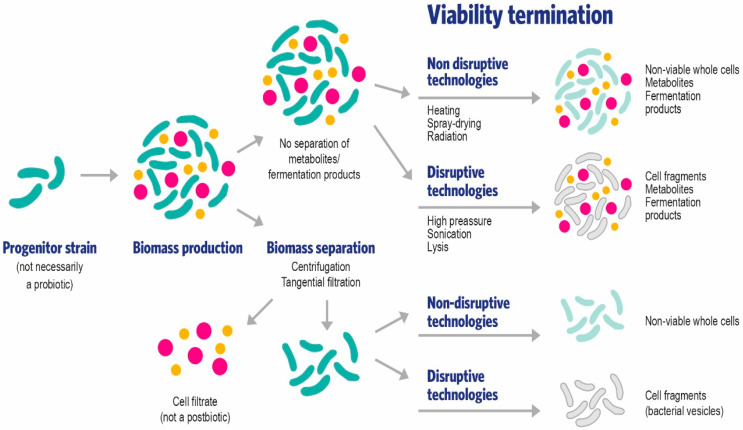
Proposed technological paths for the preparation of inanimate cultures composed of whole cells or their fragments, with and without metabolites or fermentation products, before the assessment of the postbiotic capacity through a high-quality efficacy study on the target host.

A postbiotic was defined in this work. Figure 2 depicts various ways in which an inanimate culture could be prepared before assessing it as a candidate postbiotic. Without a cautious consideration of both definitions and scopes, probiotics and postbiotics could be regarded as two sides of the same coin, with the loss of viability being the path to go from one category (probiotic) to the other (postbiotic). However, a dead probiotic in a food does not make it a postbiotic food even if some strains may be used in both categories. For instance, *Bifidobacterium bifidum* MIMBb75 significantly alleviates irritable bowel syndrome in its viable form [22] and also as a heat-inactivated culture [23]. A viable culture of *Lactobacillus gasseri* CP2305 was able to enhance quality of life and clinical symptoms in patients with irritable bowel syndrome [27], and as an inactivated culture was able to positively regulate gut environment and function [28]. In a similar fashion, *Akkermansia muciniphila* ATCC BAA-835 improved several metabolic parameters in obese and overweight volunteers when administered alive or in its pasteurized, inanimate form. Despite these examples, a microbe can only be regarded as a postbiotic if it is properly characterized, deliberately prepared with a reproducible method for inactivation, and shown to confer a health benefit. A probiotic microorganism that gradually loses cell viability over the shelf life of the food does not gradually become a postbiotic; it is simply a probiotic food that, if formulated properly, will deliver an efficacious dose of live cells until the end of its shelf life.

Although yoghurt is defined by the *Codex Alimentarius* [29] to include live starter cultures, a product known as ambient yoghurt has emerged. Ambient yoghurt is conventional yoghurt that is subjected to thermal treatment in order to inactivate starter cultures. As such, ambient yoghurt is more correctly named “yoghurt-based product for ambient distribution”. These products do not need to be stored or distributed in cold conditions. Ambient yoghurt is experiencing growth in popularity, spreading across Asia and into Africa and Latin America. In China, ambient drinking yoghurt first appeared on the market in 2010, and this fast-growing market amounts to almost 2.5 billion liters and now accounts for almost 50% of total yoghurt sales (https://www.tetrapak.com/insights/cases-articles/the-rise-of-ambient-yoghurt, accessed on 22 February 2022). The viability of starter bacteria is emphasized in food standards and regulations for yoghurt and fermented milks. Cultured milks that do not deliver viable cultures have longer shelf lives and easier storage, but beneficial health effects conferred by non-viable microbes, beyond those conferred by the micro- and macro-nutrients, therein remain to be demonstrated. Most health benefits reported for fermented milks have been documented using viable bacteria, and data on non-viable preparations are often limited. One study showed that the treatment of acute gastro-enteritis demonstrated clinical efficacy in shortening the duration of diarrhea using fermented milks with both viable and non-viable forms [30]. In this sense, ambient yoghurt may fit the definition of a postbiotic food once health benefits are properly demonstrated through efficacy trials.

Infant formulas are another example of foods used to deliver probiotics, prebiotics, synbiotics and also postbiotics [31]. In particular, *Bifidobacterium breve* C50 and *Streptococcus thermophilus* 065 have been used to produce a fermented infant formula whose microbes are inactivated by spray drying after fermentation. The infant formula carries inanimate microbes and fermentation products. A series of clinical studies in children demonstrated its safety and postbiotic properties, such as modulation of the gut microbiota to be closer to that of breastfed infants [32], reduction in the severity of acute diarrhea [33], improved inflammatory and immune markers, which might be related to some features of gastrointestinal tolerance [34], reduction in digestive and respiratory events in infants at high risk of allergy [35] and the induction of positive effects on thymus size and stool pH in healthy term infants [36]. In a broader approach, postbiotics were systematically reviewed in relation to the prevention and treatment of common infectious diseases among children younger than 5 years. Seven RCTs involving 1740 children met the inclusion criteria. For therapeutic trials, supplementation with heat-killed *Lactobacillus acidophilus* LB reduced the duration of diarrhea. For preventive trials, the pooled results from two RCTs showed that heat-inactivated *L. paracasei* CBA L74 reduced the risk of diarrhea, pharyngitis and laryngitis [37].

Food supplements form a promising category for the development of new postbiotic products as the lack of viability may offer longer stability to postbiotic products compared to probiotic food supplements. Tablets containing heat-inactivated *L. gasseri* CP2305 were administered for 4 weeks to patients with irritable bowel syndrome, resulting in improved subjective and objective symptoms [27]. The same tablets were used in a longer-term study (24 weeks), which improved chronic stress in healthy young adults preparing themselves for the national examination for medical practitioners [38]. Capsules containing 10^9^ cells of non-viable *B. bifidum* MIMBb75 were administered for 8 weeks to patients diagnosed with irritable bowel symptoms according to Rome III criteria. The inanimate culture was able to substantially alleviate symptoms in a real-life setting [23].

The concept of postbiotics will likely broaden the spectrum of microbes used for functional purposes. Species other than those belonging to the traditionally safe genus *Bifidobacterium* or the family Lactobacillaceae, which could not be administered live due to concerns about their safety, have been explored as potential postbiotics. For instance, the safety of heat-killed *Mycobacterium manresensis* was assessed as a novel food pursuant to EU regulation 2015/228330, and this inanimate culture showed potential against the development of active tuberculosis in a pilot human study [39].

## 5. Conclusions

Evidence is accumulating that inanimate microorganisms and/or their components are able to confer health benefits when administered in adequate amounts to a host. There is also research to support the concept that microbe-derived metabolites administered to a host may drive some beneficial physiological effects. Postbiotics pull together these two aspects of microbial influence on health into products that can be developed as foods, therapeutics and other product types to be administered for health outcomes. Mechanistic research points to important microbe-derived small molecules such as neurochemicals, short-chain fatty acids, defensins, bacteriocins and others that are produced in situ that likely mediate the many roles colonizing microbes exert on physiological function. However, this microbial activity is distinct from postbiotics, which must be administered to a host. Different terms have been used over the years to address this evolving area of research. To facilitate communication to health professionals, the industry, regulators and the general public, uniting under one, well-defined, consensus definition, make clear what fits and what does not fit within the postbiotic category. Further, it will allow better tracking of scientific papers for future systematic reviews and meta-analyses on the topic.

It is clear that most probiotic products contain inanimate microorganisms, but this does not make them ‘postbiotics’. Probiotic products are correctly considered to be probiotics as long as they are able to deliver the necessary dose of live cells to confer the expected health benefit. Inanimate cells in probiotic products may contribute to the health benefit delivered, but few have been studied, and their contribution is not clear so far. There is a need for future research to understand:

(1) Which inanimate microbes, with or without associated metabolites, are able to confer a health benefit;
(2) What mechanisms are driving the benefits;
(3) What role inanimate microbes contained in probiotic products may play in driving health benefits.

The ISAPP definition for postbiotics is not restricted to microbes that produce metabolites in the gut, is comprehensive enough to allow the development of postbiotics from different microorganisms and encompasses products targeting non-gut anatomical sites. Further, this definition encourages innovation in a promising area for companion or production animals and plant or human health.

## Figures and Tables

**Table 1:** Definitions of ‘biotics’ family of substances put forward by consensus panels convened by ISAPP. Note that all substances have been defined in a manner that does not restrict target host, target benefit, regulatory category, site of action on the body or specific mechanism of action. All substances must be safe for their intended use and properly identified/characterized, with a documented health benefit. Adapted from Probiotics, Prebiotics, Synbiotics, Postbiotics and Fermented Foods – defined, © 2021, International Scientific Association for Probiotics and Prebiotics. https://isappscience.org/definitionsinfographic/ (accessed on 22 February 2022).

Term (Example)	Definition	SIMPLE Way to Conceptualize	Note
Probiotic (*Bifidobacterium animalis* subsp. *lactis* BB-12 [2])	Live microorganisms that, when administered in adequate amounts, confer a health benefit on the host [3]	Live microbes that are beneficial for the host health	Identity must be confirmed through genome sequencing. An efficacious dose of viable probiotics must be preserved through the end of shelf life.
Prebiotic (Inulin, FOS, or GOS [4])	A substrate that is selectively utilized by host microorganisms conferring a health benefit on the host [5]	“Food” for beneficial microbes residing in or on the host that provide a health benefit	Not all fibers are prebiotics. Candidate prebiotics include substances such as polyphenols, which are not fibers.
Synbiotic (*B. lactis* BB-12 + inulin [6])	A mixture comprising live microorganisms and substrate(s) selectively utilized by host microorganisms that confers a health benefit on the host [7]	Probiotic + Prebiotic, defined as a complementary synbiotic	Two types of synbiotics have been defined: complementary and synergistic. A synergistic synbiotic contains a live microbe (not necessarily a proven probiotic) and a substrate (not necessarily a proven prebiotic) that it can use for growth.
Postbiotic (heat-killed *Akkermansia mucinophila* ATCC BAA-835 [8])	Preparation of inanimate microorganisms and/or their components that confers a health benefit on the host [1]	Intact non-viable microbes or cell fragments, with or without metabolites that provide a health benefit	Purified metabolites do not qualify as postbiotics

**Table 2:** Published definitions of postbiotics (adapted from [12], CC BY 4.0 (https://creativecommons.org/licenses/by/4.0/, accessed on 22 February 2022)).

Definition	Microbial Cells/Cell Components Included?	Metabolites Included in the Absence of Cells/Cell Components?	Scope Limited to Substances Produced by a Probiotic?	Health Benefit Required?	Is In Situ Production of ‘Postbiotic’ Sufficient?
Any factor resulting from the metabolic activity of a probiotic or any released molecule capable of conferring beneficial effects to the host in a direct or indirect way [10]	No	Yes	Yes	No	Yes
Soluble factors (products or metabolic byproducts), secreted by live bacteria, or released after bacterial lysis, such as enzymes, peptides, teichoic acids, peptidoglycan-derived muropeptides, polysaccharides, cell surface proteins, and organic acids [13]	No	Yes	No	No	Yes
Compounds produced by microorganisms released from food components or microbial constituents, including non-viable cells that, when administered in adequate amounts, promote health and well-being [14]	Yes (not required)	Yes	No	Yes	No
Non-viable metabolites produced by probiotics that exert biological effects on the hosts [15]	No	Yes	Yes	No *	Yes
Non-viable bacterial products or metabolic byproducts from probiotic microorganisms that have positive effects on the host or microbiota [16]	No	Yes	Yes	No **	Yes
Functional bioactive compounds, generated in a matrix during fermentation, which may be used to promote health [17]	No	Yes	No	Yes	No
Preparation of inanimate microorganisms and/or their components that confers a health benefit on the host [1]	Yes (required)	No	No	Yes	No

* Biological, but not health effects, stipulated. ** A health benefit is not specifically stipulated.

## Data Availability

No new data were created or analyzed in this study. Data sharing is not applicable to this article.
